# Efficacy of 405 nm Light-Emitting Diode Illumination and Citral Used Alone and in Combination for Inactivation of *Vibrio parahaemolyticus* on Shrimp

**DOI:** 10.3390/foods11142008

**Published:** 2022-07-07

**Authors:** Yingying Zhang, Shuo Wang, Du Guo, Zhiyuan Liu, Jianxue Gao, Xiangjun Zhan, Yutang Wang, Chao Shi, Xiaodong Xia

**Affiliations:** 1 College of Food Science and Engineering, Northwest A&F University, Xianyang 712100, China; zhang_yingying@nwafu.edu.cn (Y.Z.); wangshuoddu@126.com (S.W.); duguo2911@163.com (D.G.); lzy022169@126.com (Z.L.); gaojianxue@xjtu.edu.cn (J.G.); zxj666@nwafu.edu.cn (X.Z.); wyt991023@nwsuaf.edu.cn (Y.W.); meilixinong@nwsuaf.edu.cn (C.S.); 2 School of Food Science and Technology, National Engineering Research Center of Seafood, Dalian Polytechnic University, Dalian 116034, China

**Keywords:** 405 nm LED, *Vibrio parahaemolyticus*, citral, shrimp, cell membrane

## Abstract

*Vibrio parahaemolyticus* is a widely distributed pathogen, which is frequently the lead cause of infections related to seafood consumption. The objective of the present study was to investigate the antimicrobial effect of the combination of 405 nm light-emitting diode (LED) and citral on *V. parahaemolyticus*. The antimicrobial effect of LED illumination and citral was evaluated on *V. parahaemolyticus* not only in phosphate-buffered saline (PBS) but also on shrimp. Quality changes of shrimp were determined by sensory evaluation. Changes in bacteria cell membrane morphology, cell membrane permeability, cell lipid oxidation level, and DNA degradation were examined to provide insights into the antimicrobial mechanism. The combination of LED treatments and citral had better antimicrobial effects than either treatment alone. LED combined with 0.1 mg/mL of citral effectively reduced *V. parahaemolyticus* from 6.5 log CFU/mL to below the detection limit in PBS. Combined treatment caused a 3.5 log reduction of the pathogen on shrimp within 20 min and a 6 log reduction within 2 h without significant changes in the sensory score. Furthermore, combined LED and citral treatment affected *V. parahaemolyticus* cellular morphology and outer membrane integrity. The profile of the comet assay and DNA fragmentation analysis revealed that combination treatment did not cause a breakdown of bacterial genomic DNA. In conclusion, LED may act synergistically with citral. They have the potential to be developed as novel microbial intervention strategies.

## 1. Introduction

The Gram-negative bacterium *Vibrio parahaemolyticus* is a natural constituent of fresh water, estuarine, and marine environments [1]. It is the primary cause of bacterial food poisoning in coastal areas, mostly related to contaminated seafood consumption [2]. As a widely consumed seafood, shrimps are often contaminated with *V. parahaemolyticus*. Previous studies have isolated and characterized various *V. parahaemolyticus* strains from commonly consumed fresh shrimps in domestic markets in China [3,4]. These studies provided data in support of the high risk of *V. parahaemolyticus* contamination in shrimp. After eating uncooked seafood contaminated by *V. parahaemolyticus*, people could develop acute gastroenteritis within 24 h. In addition, *V. parahaemolyticus* also causes nausea, vomiting, diarrhea, fever, and other symptoms [2].

Various methods have been used for microbial control of shrimp, such as the use of chlorine, modified atmosphere packaging, and irradiation [5]. As a very common strategy to control pathogens in seafood, the effect of chlorine to inactivate *Vibrio* cells on shrimp has been reported [6]. However, a long contact time and high concentration of chlorine are required to destroy pathogens completely, which may cause health problems in workers, such as severe respiratory tract damage and permanently reduced lung function [7]. Furthermore, the seafoods treated with chlorine often have an undesirable smell with low consumer acceptance. Therefore, an environmentally friendly and safe method should be developed as an alternative to chlorine in the seafood industry.

Recently, many research studies have been focused on the antibacterial effect of artificial light treatments with different wavelengths in agriculture and the food industry. As an alternative energy-saving light source, light-emitting diodes (LED) have attracted attraction in the microbiological safety field [8]. As a conventional approach, UV light (wavelength < 400 nm) emitted by mercury vapor lamps is becoming a worldwide recognized strategy to against potential pathogens such as bacteria, yeasts, viruses, and fungi [9]. However, UV light has shortcomings, such as its decolorization effects on food products, and its potential health risk for the operator [10,11]. Compared to traditional visible light sources, LEDs with wavelengths in the range of 400–420 nm (blue light) have several advantages, such as low cost, high durability, low energy consumption, and a significant antibacterial effect [9]. Recent studies have proven the efficacy of 405 nm LED against various foodborne pathogens, such as *Escherichia coli* O157:H7, *Salmonella* Typhimurium, *L. monocytogenes*, *S. aureus,* and *V. parahaemolyticus* [12,13,14]. In addition, 405 nm LED has exhibited promising antimicrobial effectiveness in food such as fresh-cut papaya, fresh-cut mango, and ready-to-eat fresh salmon [15,16,17]. These results have been correlated with the photodynamic inactivation of bacteria (PDI) [18]. Some intracellular molecules known as photosensitizers will absorb oxygen and produce reactive oxygen species (ROS) under 400–420 nm LED irradiation [18,19]. These in turn react with cell membranes’ lipids, enzymes, proteins, or DNA, leading to bacterial death [20].

Although the antimicrobial activity of 405 nm LED illumination has been widely studied, the limitations on the application of LED systems have also been found. In food production environments, 405 nm LED takes a long time to completely sterilize food matrices, causing food sensory and nutritional quality loss. Recently, Josewin et al. [21] demonstrated that the synergistic effects of an LED and riboflavin (100 μM) produced a 1.2 log reduction of *L. monocytogenes* on smoked salmon at 4 °C, which was more effective than either treatment alone. Thus, an additional hurdle combined with 405 nm LED illumination should be developed for the improvement of sterilization efficiency with less impact on food quality. Plant-derived compounds have been extensively used as flavoring agents and many exhibit a wide spectrum of antimicrobial activity [22]. The challenge for the practical application of plant origin antimicrobial agents is to exploit optimized low-dose combined processes to minimize toxicological effects and sensory changes [23]. Citral (C_10_H_16_O) is one of the essential oil compounds originating from herbal plants such as lemon myrtle, lemongrass, orange, lime, and bergamot [24]. It has been recognized as a safe food additive according to the National Food Safety Standard for Uses of Food Additives (GB 2760-2011) [25] and approved by the Food and Drug Administration of the United States (FDA) as a safe ingredient [26]. It has been reported to exert antimicrobial effects on *Campylobacter jejuni*, *E. coli* O157, *L. monocytogenes*, *Bacillus cereus*, *S. aureus,* and *Cronobacter sakazakii* in food matrices [27,28,29,30]. Additionally, there is increasing evidence that citral has potential anti-inflammatory and anti-corrosive effects [31,32].

These findings indicate that the use of LEDs or citral is a promising alternative to traditional preservation technologies for reducing the potential risk of food safety. However, little is known about the inactivating effect of the combination of LEDs and citral. The objective of this study was to examine the antimicrobial efficiency of the combination of 405 nm LED irradiation and citral against *V. parahaemolyticus* on fresh shrimp, as a novel application for seafood preservation. Moreover, it’s possible antimicrobial mechanism was also elucidated by investigating the cell morphology, cell membrane permeability, as well as DNA degradation.

## 2. Materials and Methods

### 2.1. Reagents

Citral (CAS 5392-40-5) was obtained from LGC Labor GmbH (Germany) at a HPLC purity of at least 99%. Citral was dissolved in dimethyl sulfoxide (DMSO; Tianjin Kemiou Chemical Reagent Co., Ltd., Tianjin, China) before being used. The final concentration of DMSO in all treated and control sample solutions was 0.5% (*v*/*v*). Fresh shrimp (*Litopenaeus vannamei*) was purchased from a local retail market in Yangling, China.

### 2.2. Bacterial Strains and Culture Conditions

*V. parahaemolyticus* ATCC 17802 was purchased from the American Type Culture Collection (ATCC, Manassas, VA, USA). *V. parahaemolyticus* 240 and *V. parahaemolyticus* 245 were seafood isolates obtained from the Food Safety and Technology Research Centre in The Hong Kong Polytechnic University. Frozen cultures were recovered from the cryovial by surface spreading on plates of Tryptone soya agar (TSA; Beijing Land Bridge Technology Co., Ltd., Beijing, China), supplemented with 3% (*w*/*v*) NaCl, and were incubated for 24 h at 37 °C. To ensure strain purity, a single colony on TSA was cultured into Tryptone Soya Broth (TSB; Beijing Land Bridge Technology Co., Ltd., China) supplemented with 3% NaCl, incubated with shaking at 130 rpm overnight at 37 °C.

### 2.3. Light-Emitting Diode (LED) Illumination System

A slightly modified version of the illumination system comprising 405 nm LED was confirmed with the method of Zheng et al. [33], with slight modifications. Initially, the top of a heat sink with a cooling fan attached with 405 nm LED was used for minimizing the thermal production of the LED chip and preventing its thermal damage. To avoid the entry of extraneous light, an ABS (acrylonitrile butadiene styrene) housing was utilized to accommodate the system assembly. During LED illumination, the bacterial suspension below the system was subjected to temperature surveillance once every 3 min with a thermocouple thermometer (Everett, WA, USA).

The light dosage was calculated based on Equation (1) [14]. Derivation of the equation was based on the fact that an inverse-square law is obeyed by the intensity of point source-irradiated light or other linear waves:

(1) $$P=2\pi I_{0}h^{2}\left(1-\frac{1}{\sqrt{1+{\left(\frac{r}{h}\right)}^{2}}}\right),$$

where *P* (mW) refers to the quantity of energy falling on the plate every second, *I*_0_ denotes the intensity reading at the plate center from a mobile LED radiometer (Linshang, Shenzhen, China), *r* (cm) represents the radius of the plate, and *h* (cm) refers to the LED (point source) height from the plate center. In this study, *I*_0_ = 3.23 mW/cm^2^, *r* = *h* = 4.5 cm, and *P* = 18.94 ± 0.05 mW/cm^2^

The computational formula for dose, *D* (energy density) (J/cm^2^), is shown below:

(2) $$D=\frac{P}{A}t,$$

where *A* represents the overall area of the plate (cm^2^), and *t* refers to the time (seconds).

### 2.4. Temperature Control

The temperature of the PBS bacterial suspension treated with LED increased due to the thermal effect of LED irradiation [14]. To monitor the temperature conditions, activated *V. parahaemolyticus* ATCC 17802, and isolates 240 and 245 were mixed equally. The ten milliliters of mixed bacterial liquid were placed in a sterile dish (d = 90 mm) and measured with a thermoelectric thermometer. The temperature changes of the fresh shrimp surface were also monitored with the thermometer (Hebei Shuangqiao Instrument Factory, Hengshui, China). The LED assembly was placed in a refrigerator at 4 °C and the temperature of bacterial suspension and fresh shrimp was recorded every 3 min for 120 min. The 405 nm LED elevated the temperature rapidly from the set temperature of 4.0 °C to 9.4 and 8.9 °C of the bacterial suspension and fresh shrimp, respectively. For this reason, non-illuminated control experiments of PBS and shrimp were carried out at 9 °C to eliminate the effect of temperature rise during the irradiation.

### 2.5. Antimicrobial Activity of LED Combined with Citral against V. parahaemolyticus in PBS

The three working cultures of *V. parahaemolyticus* (ATCC 17802, isolate 240, isolate 245) were centrifuged (Eppendorf, Hamburg, Germany) at 8000× *g* for 5 min following washing twice with PBS (pH 7.2). The resultant pellets were re-suspended in PBS to make up an initial population of approximately 10^6^ CFU/mL. Three working cultures were mixed to obtain the final bacterial suspension.

In the LED and the LED plus 0.1 mg/mL citral groups, 10 mL of the bacterial suspension was placed in a glass petri dish (d = 60 mm) and then illuminated for 1 h at 4 °C. Experiments of control and citral groups were performed with the same amount of bacterial suspension but placed in an incubator at 9 °C. An aliquot of 100 μL was withdrawn at 0, 2, 5, 10, 20, 30, and 60 min, and these aliquots were plated onto TSA supplemented with 3% NaCl, followed by incubation at 37 °C for 24 h and counting.

### 2.6. Antibacterial Effect of LED Combined with Citral against V. parahaemolyticus on Shrimp

One hundred fresh shrimps were collected from a retail market, frozen, and stored in a −20 °C refrigerator. Individual shrimp had an average weight of 15.0 g and a length of 13.0 cm. The difference of shrimp length and weight of all experimental samples did not exceed ±10%. Briefly, shrimp with head and shell were washed with sterile water and immersed in 0.02% (*v*/*v*) NaClO disinfectant for 10 min under aseptic conditions. Then, residual NaClO was removed from shrimp by washing twice with sterile water and the surface of the samples was wiped with a sterilized paper towel. Bacterial suspension of *V. parahaemolyticus* strains ATCC 17802, 240, and 245 was prepared according to the method in Section 2.5. Citral was dissolved in DMSO, then 50 μL of citral solution was added into the bacterial solution of the LED plus citral group and the citral group. The concentration of citral in the bacterial solution of both groups was 0.1 mg/mL. The same dose of DMSO was added to the control group and the LED treatment. The shrimp samples were then immersed into bacterial suspensions and mixed, followed by being placed in sterile petri dishes. The dishes were placed under LED illumination at 4 °C, while the treatments without illumination were set at 9 °C to offset the rise in temperature generated by LED irradiation. Samples were withdrawn at 0, 5, 10, 20, 60, and 120 min post-inoculation. After vortexing for 2 min in PBS solution, aliquots of 100 μL of the dilution series were plated on TSA supplemented with 3% NaCl medium and incubated at 37 °C for 24 h.

### 2.7. Sensory Evaluation for Fresh Shrimp by Trained Panel

Fresh shrimp samples were prepared as described in Section 2.6. Shrimps of each group were treated with 405 nm LED, 0.1 mg/mL of citral, and 405 nm LED plus 0.1 mg/mL of citral for 120 min, respectively. Then, the sensory quality of shrimp samples was evaluated as described by Jeyasekaran et al. [34] with some modifications, as shown in Table S1. The 10 trained sensory assessors carried out sensory evaluation in terms of the smell, appearance, and texture of the samples, respectively. Then, the 3 parameters were added together as the overall acceptance. The total scores were between 18 points (extremely fresh) and 3 points (totally corrupted).

### 2.8. Bacterial Morphology

The cell morphology of *V. parahaemolyticus* ATCC 17802 was observed and recorded by FESEM (S-4800; Hitachi, Tokyo, Japan). The bacterial suspension of the four groups (control, LED-illuminated, 0.1 mg/mL citral, and LED-illuminated + 0.1 mg/mL citral) was washed twice with PBS buffer and added with 2.5% (*v*/*v*) glutaraldehyde solution (prepared with PBS buffer), sealed with plastic film, and fixed for 10 h at 4 °C. Suspension was washed with PBS solution and sterile water, then added with 1% osmium acid, followed by fixing for 5 h. The bacteria were gradient eluted with 30%, 50%, 70%, 80%, and 90% ethanol, followed by dissolving in ethanol. The droplets of the final suspension were added on the sterilized round cover slides and transferred into a centrifugal tube for air-drying. The slides were dried in a high vacuum, coated with gold, and observed at 20 kV.

### 2.9. Determination of Bacterial Outer Membrane Integrity

To elucidate the antimicrobial mechanism of LED illumination and citral against *V. parahaemolyticus*, bacterial outer membrane integrity was performed in accordance with the method of Shi et al. [29], with minor modifications. LIVE/DEAD^®^ BacLight Viability Kit L-7007 (Molecular Probes, Eugene, OR, USA) was used, which encompassed SYTO^®^9 (green fluorescent dye) and PI (propidium iodide, red fluorescent dye). When bacterial suspension was incubated with these nucleic acid dyes, green fluorescence was produced from the viable bacteria having intact cellular membranes, whereas red fluorescence was produced from the dead bacteria with compromised membranes. Briefly, the overnight culture of *V. parahaemolyticus* ATCC 17802 was collected and resuspended using 0.85% NaCl. For harvesting of live and dead cells, 1 mL of bacteria suspension was added separately into 20 mL of 0.85% NaCl and 20 mL of 70% isopropyl alcohol. After incubating at 25 °C for 1 h, the optical density of both samples was adjusted at 600 nm to 0.5. Bacterial suspensions were formulated by mixing varying percentages of viable cells (0, 10%, 50%, 90%, and 100%) with non-viable cells, and the standard curve was plotted. Cells treated with 405 nm LED and 0.1 mg/mL of citral for 30 min were centrifuged, followed by pipetting 100 μL of samples into 96-well black, opaque microtiter plates. Each well was pipetted with 100 μL aliquots of the 2× working staining solution, and thorough mixing proceeded. The mixture was incubated at 25 °C for 10 min in the dark. Dyes trapped inside the cells were immediately measured using InfiniteTM M200 PRO (TECAN, Mannedorf, Switzerland), and set of fluorochrome filters: excitation wavelength (485 nm), emission wavelength SYTO^®^9 (542 nm), and PI (610 nm).

### 2.10. Comet Assay

Bacterial suspensions of *V. parahaemolyticus* ATCC 17802 were prepared according to the method of Kim et al. [12] with a slight modification, using the Comet Assay Kit (Abcam ab238544, Shanghai, China). Here, 75 μL of the aliquot was pipetted on comet slides, then slides were placed at 4 °C for 15 min to fix the agarose. Thereafter, a 1 h immersion of the comet slides was accomplished in pH 10 Lysis Buffer under dark conditions at 4 °C, and then a further 30 min immersion proceeded in alkaline solution for the slides under the same conditions. Electrophoresis was performed for 20 min (12 V, 100 mA) using alkaline electrophoresis buffer. The slides were then subjected sequentially to three washings with distilled water, each for 2 min, a 10 min dehydration using 70% (*v*/*v*) chilled ethanol, drying in air, and subsequent staining with 100 μL of Vista Green DNA Dye staining solution for each well. A Leica DM6 B epifluorescent microscope (Wetzlar, Germany) was utilized in conjunction with Vista Green DNA Dye (WB, 450–480 nm) for acquisition of micrographs at a magnification of 1000×.

### 2.11. DNA Fragmentation Analysis

*V. parahaemolyticus* ATCC 17802 DNA fragmentation following the combination of 405 nm LED treatment and 0.1 mg/mL of citral was analyzed according to the method of Kim et al. [12] with a slight modification, using the TaKaRa MiniBEST Bacteria Genomic DNA Extraction Kit (TAKARA BIO INC). The purified DNA was dissolved in 150 µL of Elution Buffer. The 40 mL 1% (*w*/*v*) agarose gel solution was boiled 3 times, cooled for a moment, and added with 4 µL of GelRed nucleic acid dye. Samples with loading buffer (5:1, *v*/*v*) were electrophoresed at 120 V for 30 min. The gel was visualized with GELDOC XR+ (Bio-Rad Laboratories, Co., Ltd., Shanghai, China).

### 2.12. Statistical Analysis

Significant differences in the mean value were calculated at the 95% confidence interval (*p* < 0.05) using one-way analysis of variance (ANOVA). The Kruskal–Wallis test was used for a comparison of sensory scores. All statistical analysis was performed using the IBM SPSS statistical software (version 19.0; SPSS Inc., Chicago, IL, USA). Data were expressed as mean ± standard deviation (SD) (*n* = 6).

## 3. Results

### 3.1. Bacterial Inactivation by Combination of LED Illumination and Citral in PBS

The inactivation effects of 405 nm LED and 0.1 mg/mL of citral against *V. parahaemolyticus* in PBS were investigated and the results are shown in Table 1. The population of non-illuminated cells in the control group remained stable within 1 h, at 3.16 × 10^6^ CFU/mL. The population of citral-treated cells showed a 1.5 log (CFU/mL) reduction after 1 h. The inactivation of *V. parahaemolyticus* treated with the LED illumination and 0.1 mg/mL of citral was most effective compared to the other treatments, producing a severe reduction of 6.5 log after 5 min. The number of viable cells was lower than the detectable limit after 10 and 5 min of LED treatment and LED combined with citral treatment, respectively.

### 3.2. Bacterial Inactivation by Combination of LED Illumination and Citral on Shrimp

Shrimp were inoculated with bacterial suspension and observed for 2 h, as shown in Table 2. After 2 h of LED illumination, the bacterial population reduced about 3 log (CFU/mL). Additionally, the presence of 0.1 mg/mL of citral led to a 2 log reduction in the population of *V. parahaemolyticus* on shrimp. The inactivation of *V. parahaemolyticus* through LED illumination treatment in combination with 0.1 mg/mL of citral was most effective, and the number of bacteria decreased to 3.5 log (CFU/mL) within 20 min and fell below the detection limit (10 CFU/mL) after 2 h.

### 3.3. Sensory Evaluation for Shrimp

**Figure 1:**
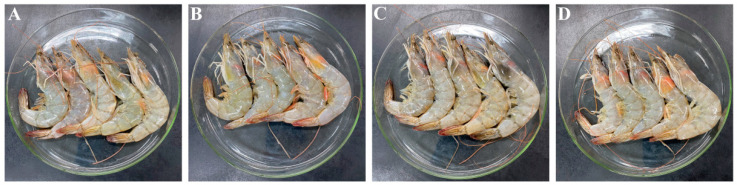
Effect of combination of 405 nm LED and 0.1 mg/mL of citral on sensory evaluation of shrimp. (**A**) Untreated, (**B**) treated with 405 nm LED irradiation for 120 min, (**C**) treated with 0.1 mg/mL of citral for 120 min, and (**D**) LED treatment combined with 0.1 mg/mL of citral for 120 min.

The effect of 405 nm LED and 0.1 mg/mL of citral on shrimp is shown in Figure 1. The overall sensory scores of shrimp with difference treatments are shown in Table 3. The statistical analysis of the overall acceptance showed no significant difference (*p* > 0.05) between samples. Although the combination of LED and the citral treatment sample had obtained lower scores for the odor attribute (5.0 ± 0.4), the overall acceptance of all shrimp samples was higher than 15 points with all treatments, indicating that the three treatments did not affect the overall sensory quality of the raw shrimp.

### 3.4. FESEM Observations

**Figure 2:**
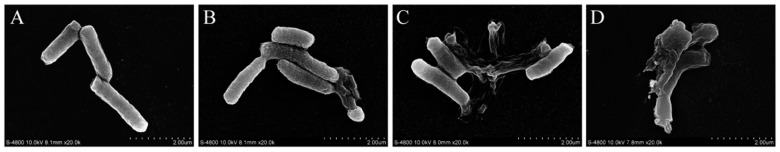
Scanning electron micrographs of *Vibrio parahaemolyticus.* (**A**) Untreated, (**B**) treated with 405 nm LED irradiation for 10 min, (**C**) treated with 0.1 mg/mL of citral for 10 min, and (**D**) LED treatment combined with 0.1 mg/mL of citral for 10 min.

As shown in Figure 2, the shape of untreated cells was rod-shaped and smooth, while LED-treated cells were ruptured after 10 min of exposure (Figure 2B). Cells showed shrinkage with the presence of 0.1 mg/mL of citral (Figure 2C). When cells were treated with LED and citral, cells showed significant shrinkage, with some cells breaking into pieces (Figure 2D).

### 3.5. Effect of 405 nm LED Illumination and Citral on Outer Membrane Integrity

**Figure 3:**
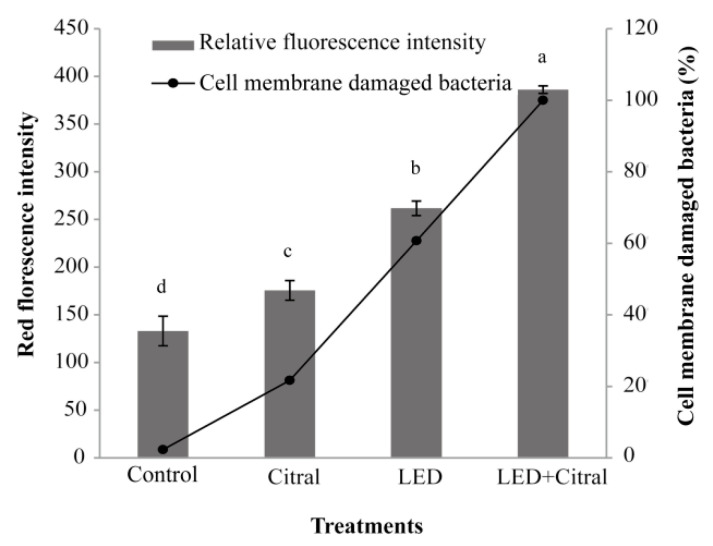
Effect of 405 nm LED illumination and 0.1 mg/mL of citral on outer membrane integrity of *Vibrio parahaemolyticus* for 30 min. Means marked with different lower-case letters are statistically different (*p* < 0.05).

The bacterial suspensions were prepared by mixing 0%, 10%, 50%, 90%, and 100% of the volume of live bacteria with the dead bacteria, respectively. The standard curve was set properly. As shown in Figure 3, LED illumination and 0.1 mg/mL of citral treatment had certain effects on the cell membrane permeability of *V. parahaemolyticus*. The relative intensity of red fluorescence of the citral group was 175 ± 9.6 (A.U), while the relative intensity of red fluorescence of the LED group was 261 ± 7.3 (A.U), and the relative red fluorescence intensity of LED and 0.1 mg/mL of citral was 385 ± 4.3 (A.U). After being treated with LED illumination combined with citral, the proportion of dead bacteria reached 100%.

### 3.6. Comet Assay

**Figure 4:**
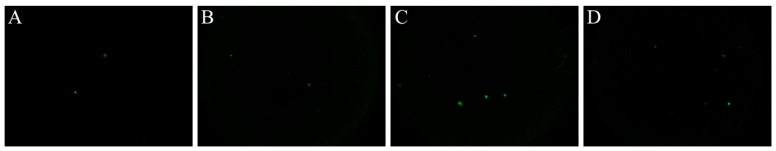
Comet assay of DNA extracted from *V. parahaemolyticus* ATCC 17802 from different treatments. (**A**) Untreated, (**B**) treated with 0.1 mg/mL of citral for 30 min, (**C**) treated with LED irradiation for 30 min, and (**D**) LED treatment combined with 0.1 mg/mL of citral for 30 min.

The comet assay was adopted for determining whether 405 nm LED irradiation and 0.1 mg/mL of citral addition would lead to DNA degradation. As shown in Figure 4, only clear heads were found in both LED-treated and untreated cells, suggesting that LED irradiation did not cause DNA breakage. Similarly, no tails (comets) were observed in citral and the LED combined with citral treatments. All single-cell electrophoresis photos observed by microscopy presented clear zones of the nucleus in cells without DNA tails.

### 3.7. DNA Fragmentation Analysis

**Figure 5:**
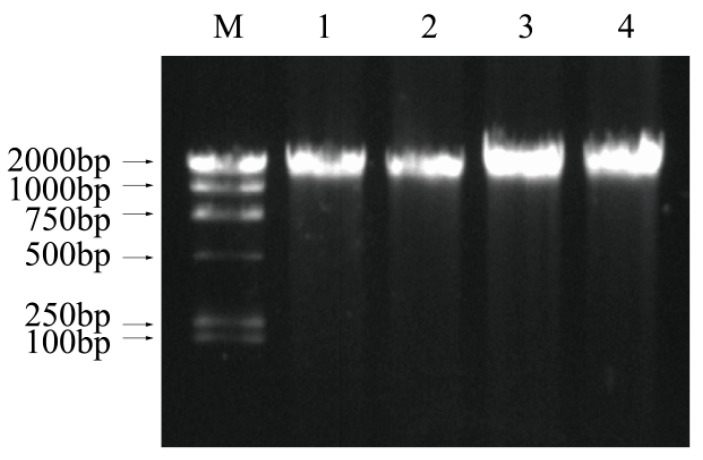
DNA fragmentation profiles of DNA extracted from *Vibrio parahaemolyticus*. Lane: M, λ/HindIII DNA marker; 1, untreated; 2, treated with 405 nm LED irradiation for 30 min; 3, treated with 0.1 mg/mL of citral for 30 min; 4, LED treatment combined with 0.1 mg/mL of citral for 30 min.

There was only one positive band present at 2000 bp in all DNA ladder profiles (Figure 5). No DNA fragments were observed, and the DNA migration bands of all samples were the same, indicating that no differences were observed in total genomic DNA among untreated, LED illumination, 0.1 mg/mL citral treatment, and the combination of LED and citral treatment cells. These results indicated that the three sterilization treatments did not induce DNA breakage in *V. parahaemolyticus*.

## 4. Discussion

The present study detected the effectiveness of 405 nm LED combined with 0.1 mg/mL of citral in inactivating *V. parahaemolyticus* on the shrimp surface to see if the LED technology has a potential to be applied to seafood preservation. Moreover, the bacterial membrane damage and DNA breakage of *V. parahaemolyticus* were determined to reveal the mechanism of the inactivation by 405 nm LED and citral.

LED with a specific wavelength (405 nm) has been proven to have a microbial inactivation effect [10,35,36]. The optimized LED sterilizing device in this study has a power of 20 W, a wavelength of 405 ± 5 nm, and was sterilized at 4 °C to simulate a practical food storage condition. Citral is a kind of edible plant-derived bacteriostatic agent whose antimicrobial action against some common pathogens has been demonstrated. Somolinos et al. [37] showed that citral at 0.2 μL/mL caused more than a 5 log CFU/mL *E. coli* cells’ reduction at pH 4.0 for 24 h. Citral also showed antimicrobial activity against *L. monocytogenes*, *C. sakazakii,* and *V. parahaemolyticus* at relatively low concentrations. The MICs of citral against *L. monocytogenes* strains ranged from 60 to 300 μg/mL [38]. The MIC of citral against *C. sakazakii* strains was 0.6 μL/mL [37]. In Guo’s study, the MICs of citral against *V. parahaemolyticus* ATCC 17802 was 0.1 mg/mL [38]. For this reason, we chose 0.1 mg/mL as an effective concentration of citral in this study. In the present study, 405 nm LED illumination combined with 0.1 mg/mL of citral inactivated 6 log CFU/mL of the populations of *V. parahaemolyticus* at 4 °C for 5 min, which could improve the efficiency of sterilization and significantly shorten the sterilization time. Additionally, the LED illumination treatment produced a severe reduction of 6 log in the population of bacteria. Similarly, Maclean et al. [10] reported that a 5 log reduction of *S. aureus* was conducted by 405 nm LED irradiation at a total dose of 36 J/cm^2^. Endarko et al. [39] also showed that *L. monocytogenes* was decreased by about 5 log during the 405 nm LED irradiation at a dose of 185 J/cm^2^. However, Endarko et al. [39] demonstrated that only slight reductions were observed in the population of *S. Enteritidis* by 1.36 log at a total dose of 739 J/cm^2^, indicating that the antibacterial efficacy of 405 nm LED might be strain-dependent.

In the present study, 405 nm LED inactivated about 3 log CFU/mL of the population of *V. parahaemolyticus* for 2 h on shrimp. It was noticed that the population of *V. parahaemolyticus* was reduced to below the detection limit from 10^6^ CFU/mL in LED, synergistic with 0.1 mg/mL of citral treatment for 2 h on the shrimp sample. It was more efficient to inactivate the bacteria on the shrimp surface than that of LED or citral alone. The effectiveness of 460 nm LED was also shown by Zheng et al. [33], where *C. sakazakii* was inactivated in powder infant milk with the concentration decreased from 8 to 1 log CFU/g. However, 460 nm LED illumination only led to significant bacterial inactivation in PBS, and did not decrease *L. monocytogenes* populations surviving on salmon [18]. The differences of temperature, acidity, and polysaccharides’ content in food matrices are probably the factors affecting the sterilization efficiency of LED and plant-derived natural components [16]. For sensory evaluation, LED illumination combined with 0.1 mg/mL of citral treatment did not affect the appearance, texture, odor, and acceptance of the shrimp sample. In terms of food quality parameters, whether LED combined with citral can be directly applied in other foods needs to be further studied.

As a selective permeation barrier around bacteria, the cell outer membrane protects bacteria from harmful substances, but allows nutrients to enter to maintain growth [40]. The results of SEM showed that *V. parahaemolyticus* cells shrink and rupture severely after 10 min treatment by LED illumination and 0.1 mg/mL of citral (Figure 2). In the LIVE/DEAD^®^ BacLight™ assay, LED combined with 0.1 mg/mL of citral treatment made the cell grinding rupture ratio 100%, eventually (Figure 3). In the present study, some cells treated with citral alone showed obvious shrinkage without cell disintegration, suggesting that citral may bind to the cell surface [41]. Consistent with a previous investigation, Shi et al. showed that citral changes the bacteria cell morphology, and affects the bacteria cell membrane by decreased intracellular ATP concentration and reduced intracellular pH and cell membrane hyperpolarization. Compared with citral treatment, LED illumination caused more severe outer membrane damage in *V. parahaemolyticus*. Maclean et al. [10] found that the molecular conformation of partial proteins and lipids in the outer cellular membranes was altered by 405 nm light, which could gradually disrupt such membranes, progressively. The membrane lipids, being one of the chief ROS targets in the oxidative stress context, can be used to explain the LED illumination-elicited disruption of cellular membranes [42]. In our postulation, the ROS generated by LED-illuminated bacteria probably interact with the unsaturated fatty acids in the bacterial membranes directly to alter the membrane components, which facilitates better penetration of the outer cellular membranes by citral, ultimately to attain a synergistic bactericidal effect.

Genomic DNA is a critical ROS target generated upon oxidative stress, such as ultraviolet light irradiation and ionizing radiation. Through generation of oxidized derivatives (e.g., 8-hydroxy-deoxyguanosine) and damage of guanine bases, the ROS may result in the DNA breakage [43]. The comet assay and DNA ladder analysis were performed to investigate whether ROS generated by 405 nm LED irradiation would lead to DNA degradation, as well as determine the effect of 0.1 mg/mL of citral on DNA breakage. In this work, no DNA tailing was observed in the comet assay, and the overall assessment of thge genomic DNA ladder revealed no difference among untreated, LED-illuminated, and 0.1 mg/mL of citral treatment cells, implying that the 405 nm LED or 0.1 mg/mL of citral probably do not lead to rupture of bacterial DNA. Similarly, Nitzan and Ashkenazi [43] also found no breakage of DNA in *E. coli* after visible light irradiation with different wavelengths (400–450, 480–550, and 600–700 nm), while cytoplasmic membrane damage was observed. Kim et al. [12,20] also reported that the illumination with 405 ± 5 nm LED did not lead to DNA breakage in *B. cereus*, *E. coli* O157:H7, *L. monocytogenes*, *S. aureus*, *S. Typhimurium*, and *S. sonnei*. Most likely, the low concentration of ROS generated by *V. parahaemolyticus* treated with LED irradiation was not sufficient to induce breakage of bacterial DNA. It might only oxidize other cellular components, such as proteins and lipids. Furthermore, the effects of LED illumination and citral on plasmid DNA in *V. parahaemolyticus* need to be confirmed in the future.

## 5. Conclusions

In conclusion, the present study evidenced the antibacterial effect of 405 nm LED combined with citral on *V. parahaemolyticus* under refrigerated conditions. The results reveal that a 405 nm LED illumination combined with 0.1 mg/mL of citral could effectively reduce the number of *V. parahaemolyticus* in both PBS and fresh shrimp than either treatment individually. Besides, the findings indicate that 405 nm LED and 0.1 mg/mL of citral treatment had no significant effect on the sensory quality of the food sample, destroyed the cell outer membrane morphology of *V. parahaemolyticus,* and damaged the outer membrane integrity. In addition, neither LED nor citral induced genomic DNA fragmentation in *V. parahaemolyticus*. Thus, 405 nm LED in combination with 0.1 mg/mL of citral might be a promising technology in eliminating *V. parahaemolyticus* in stored shrimp. The present study suggests the potential for using 405 nm LED combined with citral as a non-thermal and green technology for the control of pathogenic bacteria in the food matrix. In the future, studies are expected to better simulate a practical scenario with LED illumination and citral treatment in different food matrices.

## Figures and Tables

**Table 1:** Inactivation of *Vibrio parahaemolyticus* by combination of 405 nm LED illumination and citral in PBS.

Time (min)	*Vibrio parahemolyticus* (log CFU/mL)			
	Control	LED	Citral	LED + Citral
0	6.56 ± 0.08 ^a^	6.56 ± 0.08 ^a^	6.56 ± 0.08 ^a^	6.56 ± 0.08 ^a^
2	6.61 ± 0.10 ^a^	5.54 ± 0.12 ^a^	6.71 ± 0.10 ^a^	2.70 ± 0.01 ^a^
5	6.49 ± 0.23 ^a^	4.51 ± 0.19 ^b^	6.15 ± 0.33 ^a^	ND ^c^
10	6.28 ± 0.27 ^a^	ND ^c^	6.03 ± 0.25 ^b^	ND ^c^
20	6.37 ± 0.12 ^a^	ND ^c^	5.58 ± 0.22 ^b^	ND ^c^
30	6.52 ± 0.41 ^a^	ND ^b^	5.54 ± 0.27 ^a^	ND ^b^
60	6.26 ± 0.22 ^a^	ND ^c^	5.03 ± 0.30 ^b^	ND ^c^

ND: Not detected. Different letters indicate significant (*p* < 0.05) differences.

**Table 2:** Inactivation of *Vibrio parahaemolyticus* by combination of 405 nm LED illumination and citral on shrimp.

Time (min)	*Vibrio parahemolyticus* (log CFU/mL)			
	Control	LED	Citral	LED + Citral
0	6.02 ± 0.18 ^a^	6.02 ± 0.18 ^a^	6.02 ± 0.18 ^a^	6.02 ± 0.18 ^a^
5	6.02 ± 0.18 ^a^	4.63 ± 0.25 ^ab^	5.83 ± 0.23 ^b^	4.65 ± 0.38 ^b^
10	5.84 ± 0.26 ^a^	4.43 ± 0.19 ^a^	5.76 ± 0.22 ^b^	4.30 ± 0.15 ^b^
20	5.81 ± 0.19 ^a^	3.12 ± 0.09 ^a^	5.60 ± 0.24 ^b^	2.45 ± 0.10 ^b^
60	6.07 ± 0.10 ^a^	2.75 ± 0.09 ^a^	5.28 ± 0.07 ^b^	1.78 ± 0.33 ^c^
120	5.90 ± 0.18 ^a^	2.53 ± 0.02 ^b^	4.35 ± 0.21 ^c^	ND ^d^

ND: Not detected. Different letters indicate significant (*p* < 0.05) differences.

**Table 3:** Sensory evaluation of fresh shrimp with LED illumination, 0.1 mg/mL of citral, and LED plus 0.1 mg/mL of citral treatment for 120 min.

Treatments	Odor	Appearance	Texture	Overall Acceptance
Control	5.3 ± 0.6 ^a^	5.3 ± 0.5 ^a^	5.3 ± 0.8 ^a^	15.9 ± 1.3 ^a^
LED	5.5 ± 0.5 ^a^	5.3 ± 0.6 ^a^	5.2 ± 0.7 ^a^	16.0 ± 1.3 ^a^
Citral	5.6 ± 0.5 ^a^	5.0 ± 0.8 ^a^	5.2 ± 0.6 ^a^	15.8 ± 1.4 ^a^
LED + Citral	5.0 ± 0.4 ^a^	5.3 ± 0.6 ^a^	5.6 ± 0.5 ^a^	15.9 ± 1.0 ^a^

Mean ± standard deviation (*n* = 10). Same letters indicate no significant differences (*p* > 0.05).

## Data Availability

The data presented in this study are available upon request from the corresponding author.

## Supplementary Materials

The following are available online at https://www.mdpi.com/article/10.3390/foods11142008/s1, Figure S1: Light-emitting diode (LED) illumination system. Table S1: The grading standard for fresh shrimp sensory evaluation.
