# Supplementary material for: Efficacy of 405 nm Light-Emitting Diode Illumination and Citral Used Alone and in Combination for Inactivation of Vibrio parahaemolyticus on Shrimp
Source: Foods. 2022 Jul 7;11(14):2008. doi: 10.3390/foods11142008 (PMC9324625; doi:10.3390/foods11142008)
Supplement: Supplementary file 1 [file foods-11-02008-s001.zip › foods-1796936-SI.pdf]

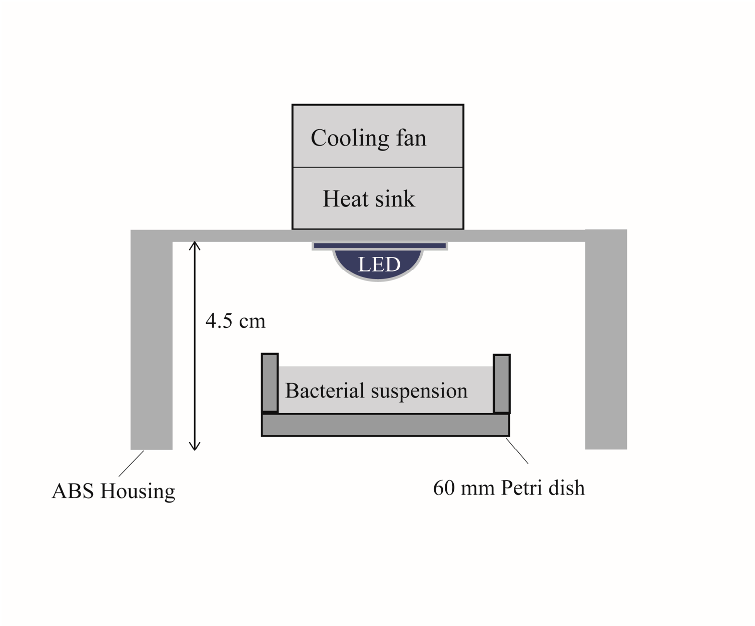


**Figure S1** Light-emitting diode (LED) illumination system.

**Table S1** The grading standard for fresh shrimp sensory evaluation

| Odor | Appearance | Texture | Score |
|---|---|---|---|
| Seaweedy, characteristic of the species | Head completely attached to body, head, body and tail with characteristic color. | Firm, elastic, hard shell/body | 6 |
| Slight characteristic odor of the species | The color of head, body and tail has slightly faded | Slightly soft body/shell | 5 |
| Slightly ammoniacal | Slight loose carapace, head with slight blackening; light black spot appear in the body and tail | Slightly soft body/shell | 4 |
| Urea, slightly fishy | Head almost completely black and slightly loose; tail exhibiting marked discoloration | Soft body and shell | 3 |
| Ammoniacal, sulfide | Head slightly detached from body and almost completely black; body and tail black in color | Soft tissue and yellow flesh | 2 |
| Strong sulfide, urea, fecal, strong ammoniacal | Head completely detached from body and completely black; body and tail dark black in color | Mushy, very soft papery shell | 1 |
